# Z-ring Structure and Constriction Dynamics in *E. coli*

**DOI:** 10.3389/fmicb.2017.01670

**Published:** 2017-09-11

**Authors:** Pramod Kumar, Amarjeet Yadav, Itzhak Fishov, Mario Feingold

**Affiliations:** ^1^Department of Physics, Ben-Gurion University of the Negev Beer Sheva, Israel; ^2^The Ilse Katz Center for Nanotechnology, Ben-Gurion University of the Negev Beer Sheva, Israel; ^3^Department of Life Sciences, Ben-Gurion University of the Negev Beer Sheva, Israel

**Keywords:** bacterial division, Z-ring, optical tweezers, fluorescence microscopy, sub-pixel measurements

## Abstract

The Z-ring plays a central role in bacterial division. It consists of FtsZ filaments, but the way these reorganize in the ring-like structure during septation remains largely unknown. Here, we measure the effective constriction dynamics of the ring. Using an oscillating optical trap, we can switch individual rod-shaped *E. coli* cells between horizontal and vertical orientations. In the vertical orientation, the fluorescent Z-ring image appears as a symmetric circular structure that renders itself to quantitative analysis. In the horizontal orientation, we use phase-contrast imaging to determine the extent of the cell constriction and obtain the effective time of division. We find evidence that the Z-ring constricts at a faster rate than the cell envelope such that its radial width (inwards from the cytoplasmic membrane) grows during septation. In this respect, our results differ from those recently obtained using photoactivated localization microscopy (PALM) where the radial width of the Z-ring was found to be approximately constant as the ring constricts. A possible reason for the different behavior of the constricting Z-rings could be the significant difference in the corresponding cell growth rates.

## Introduction

The investigation of bacterial cell division is imperative both for understanding the fundamentals of the life cycle of microorganisms and for numerous applications to biomedical problems. The core of the division machinery is the so-called Z-ring, formed from polymers of the tubulin-like protein FtsZ, and acting as an internal scaffold that correctly localizes synthetic enzymes (Margolin, 2005; Dajkovic and Lutkenhaus, 2006; Erickson et al., 2010). Moreover, it was suggested that FtsZ leads the septal peptidoglycan building machinery driving septum constriction (Bisson-Filho et al., 2017; Yang et al., 2017). Crystallographic, phylogenetic, and biochemical evidence indicate that FtsZ is the prokaryotic ancestor of tubulin (Erickson, 1997; Lowe and Amos, 1998). FtsZ contains a highly conserved N-terminus and a highly conserved C-terminal tail that are connected by a region of variable sequence and length. FtsZ polymerization *in vivo* and *in vitro* requires the N-terminus but not the C-terminus (Ma et al., 1996; Yu and Margolin, 1997). The C-terminus is important for interactions with other FtsZ molecules and other proteins such as FtsA and ZipA (Ma et al., 1996). In addition to Mg^2+^, the GTPase activity of FtsZ is dependent on FtsZ protein concentration, suggesting that GTPase activity is coupled to assembly (de Boer et al., 1992; RayChaudhuri and Park, 1992; Mukherjee et al., 1993; Lu et al., 1998; Sossong et al., 1999; Salvarelli et al., 2011). These data are also supported by the FtsZ crystal structure, which revealed an active site shared by two FtsZ monomers.

Z-rings on the cytoplasmic membrane have been observed by both immunogold electron microscopy (Bi and Lutkenhaus, 1991) and fluorescence microscopy (Addinall and Lutkenhaus, 1996; Levin and Losick, 1996; Ma et al., 1996). GFP-tagged FtsZ expressed at low levels co-polymerizes with native FtsZ in the cell without inhibition of cell division, allowing to study its localization and dynamic behavior in living cells. As the septum grows inward, the Z-ring contracts at its leading edge until cytokinesis is nearly complete, at which time FtsZ disperses, perhaps by looping out subunits from the central core (Sun and Margolin, 1998). Under certain conditions, FtsZ can form spirals and arcs that successfully invaginate to form asymmetric septa (Addinall and Lutkenhaus, 1996). FtsZ does not seem to interact directly with membrane phospholipids *in vivo*. Instead, it binds to the membrane only via a complex with ZipA and/or FtsA. Positioning of the Z-ring in the cell, and consequently of the division site, is determined by two regulatory systems—MinCDE (see Rothfield et al., 2005; Lutkenhaus, 2007 for reviews) and nucleoid occlusion (Wu and Errington, 2004; Bernhardt and de Boer, 2005).

**Figure 1:**
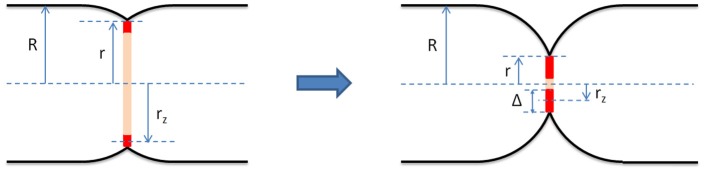
Geometrical model of the bacterial septum and the Z-ring (red) at early **(Left)** and late **(Right)** stages of constriction. It assumes that before the onset of division the cell shape is that of a cylinder with two hemispherical caps. The cell radius, *R*, is defined as the radius of the cylindrical section of the cell enclosed by the cytoplasmic membrane. We assume that *R* is constant throughout the septation process. During division, the septum consists of two incomplete hemispheres joined at midcell. We define the constriction radius, *r*, as the radius of the circle joining the two septal incomplete hemispheres. The Z-ring is modeled as a wide ring centered in the septal plane and attached to the cytoplasmic membrane. Its radius, *r*_*z*_, is measured from the middle of its radial width to the long cell axis and its radial width, Δ, is twice the difference between *r* and *r*_*z*_, Δ = 2(*r* − *r*_*z*_). Our results indicate that for fast growing *E. coli* cells the radial width of the Z-ring, Δ, grows during septation.

Z-ring dynamics *in vivo* was monitored by wide-field fluorescence microscopy (Sun and Margolin, 1998; Thanedar and Margolin, 2004; Monahan et al., 2009) and other related methods (Stricker et al., 2002). One of the important results of these works is that FtsZ displays a highly dynamic behavior, oscillating along the cell presumably driven by the regulatory MinCDE system. Spatially, these oscillations follow an extended helically shaped pattern. In *B. subtilis*, it was suggested that the helix-to-ring transition of FtsZ is promoted by ZapA, stimulating lateral FtsZ association (Monahan et al., 2009). Fluorescence recovery after photobleaching (FRAP) was also used to study the Z-ring dynamics showing a fast exchange of FtsZ between the ring and the cytoplasm (Stricker et al., 2002). Moreover, Optical Tweezers assisted imaging allowed to view the Z-ring as a circular structure (axial view) and measure its width inwards from the cytoplasmic membrane, Δ (Figure 1) (Carmon et al., 2012, 2014). Prior to the onset of constriction, it was shown that Δ is about 100 nm. This result lead to the suggestion that the Z-ring consists of a random network of FtsZ filaments (Piro et al., 2013) which, in turn, is consistent with results from polarization microscopy indicating that the FtsZ filaments in the ring are randomly oriented (Si et al., 2013).

More recently, the Z-ring was also studied using a wide range of super-resolution microscopy techniques. Fu et al. used photoactivated localization microscopy (PALM) showing that in *E. coli* the ring has a helical structure along the cytoplasmic membrane on a sub-optical resolution length scale (Fu et al., 2010). A similarly structured Z-ring was also observed in *B. subtilis* using stimulated emission depletion (STED) (Jennings et al., 2010). Biteen et al. were the first to obtain 3D super-resolution images of the Z-ring using astigmatism to determine the height of individual fluorophores in a PALM system (Biteen et al., 2012). Imaging the axial view of the Z-ring in pre-divisional *C. crescentus* cells they show that it has a significant radial width, consistent with the results of Carmon et al. (2012, 2014). Another 3D super-resolution microscopy approach, 3D structured illumination microscopy (3D-SIM), also allows obtaining an axial view of the Z-ring (Strauss et al., 2012; Rowlett and Margolin, 2014). 3D-SIM imaging revealed that the Z-ring in both *B. subtilis* and *E. coli* is inhomogeneous along its circumference displaying 120–200 nm gaps and a bead-like structure. Astigmatism 3D-PALM (Holden et al., 2014) and 2D-PALM of vertically immobilized cells (Jacq et al., 2015) were used to show that the Z-rings of *C. crescentus* and *S. pneumoniae*, respectively, exhibit a similar structure.

3D-SIM and 3D-PALM were also used to monitor the dynamics of the ring structure on the different time scales (Strauss et al., 2012; Holden et al., 2014; Coltharp et al., 2016). Strauss et al. (2012) have shown that the distribution of FtsZ along the Z-ring circumference in both *B. subtilis* and *S. aureus* significantly changes within 10 s. This behavior was observed for cells before the onset of constriction, during constriction and at a non-permissive temperature where cell division is inhibited. While providing much better resolution than 3D-SIM, 3D-PALM requires relatively long acquisition times (e.g., 80 s; Holden et al., 2014) and may lead to some photodamage, limiting its use for single cell time-lapse microscopy. Instead, 3D-PALM was used to obtain the effective dynamics of Z-ring constriction (Holden et al., 2014; Coltharp et al., 2016). The ring geometry was measured for a set of cells that were at different stages in the division process. For both *C. crescentus* and *E. coli* cells, it was shown that the radial and the longitudinal widths of the ring remain approximately constant until a certain stage of the constriction when the Z-ring rapidly collapses. To determine the time along the cell cycle for each individual cell, Holden et al. used a synchronous population, measuring time from the onset of synchrony till the imaging of the live cell (Holden et al., 2014). In contrast, Coltharp et al. used interferometric PALM (iPALM) to image the Z-ring in fixed *E. coli* (Coltharp et al., 2016). In their approach, the various ring characteristics are measured as a function of the corresponding ring diameter, that, in turn, is calibrated to indicate the effective time along the cell division period. In both Holden et al. (2014) and Coltharp et al. (2016) the effective time is obtained as a population average and the timing variability between the cells in the population is only partially accounted for. This may explain the large fluctuations in their single cell data for the radial and longitudinal widths of the Z-ring, contrasting with the high precision of 3D-PALM imaging.

Another important observation of the recent 3D-PALM studies of Z-ring constriction is that most of the rings that were imaged were incomplete and a significant fraction appeared as arcs shorter than semicircles (Holden et al., 2014; Coltharp et al., 2016). A possible reason for this behavior might be the fact that in both studies cells were grown in minimal media corresponding to long generation times, e.g., 181 min and 193 min in *E. coli* (Coltharp et al., 2016). In this slow growth regime, the amount of FtsZ available for Z-ring formation may be limited leading to strongly inhomogeneous rings. This seems consistent with the fact that there is much less FtsZ in slow growing than in fast growing cells (Link et al., 1997; Lu et al., 1998; Rueda et al., 2003; Ishihama et al., 2008; Vendeville et al., 2011). Specifically, there are ~300 FtsZ proteins in low growth rate *E. coli* cells (generation time > 100 min) (Link et al., 1997) and 3,000–15,000 in high growth rate *E. coli* (generation time ≃ 20 min) (Lu et al., 1998; Rueda et al., 2003). Another result of Coltharp et al. (2016) that, following the onset of constriction, the cylindrical cell segment stops growing and the only contribution to cell elongation is from the new cap region may also provide support for such scenario. It has been suggested that the ending of cylindrical growth is due to the competition with the growth of the new caps over the limited resources of peptidoglycan precursor materials (Begg et al., 1990; Lleo et al., 1990), analogously to the limit on the amount of FtsZ. In contrast, for fast growing cells it was shown that cylindrical growth continues throughout constriction with only a moderate slowdown, ~30%, from its predivisional rate (Reshes et al., 2008a,b).

To test whether the behavior of the Z-ring is different for *E. coli* cells in the high growth rate regime, we study the dynamics of Z-ring constriction for cells growing in Luria broth at 37°C. Although in our imaging approach we use conventional optical microscopy, optical trapping allows us to view the Z-ring as a circular structure without the need of image reconstruction. This feature of our system together with a combination of both spatial and temporal averaging leads to measurements of the Z-ring radius that are of comparable accuracy to those obtained using iPALM (Coltharp et al., 2016).

Specifically, we use optical trapping to image the Z-ring while the *E. coli* cell is oriented with its long axis either in the imaging plane or perpendicular to it. For each orientation, first the cell is imaged using fluorescence to view the GFP labeled Z-ring and next with phase contrast microscopy to obtain the cell shape. Recently, we have used this approach to measure the width of the Z-ring in unconstricted *E. coli* (Carmon et al., 2012, 2014). Here, we analyze constricting cells at different stages of the division process. Since exposure to the trapping laser of more than ~20 s interferes with normal cell growth (Neuman et al., 1999; Ayano et al., 2006), we cannot measure the ring constriction dynamics of individual cells (see Supplementary Material). Instead, we use the measured septal width to obtain an effective time of division, *t'*, for each of the individual cells (Reshes et al., 2008a,b). Together with the measured radius of the Z-ring, *r*_*z*_, this leads to the effective Z-ring constriction dynamics, *r*_*z*_(*t'*). We find that the Z-rings of fast growing *E. coli* appear to be much more uniform along their circumference than those in Holden et al. (2014) and Coltharp et al. (2016). Moreover, we present evidence that Z-rings constrict at a faster rate than the cell envelope and their radial width grows during septation.

## Materials and methods

### Bacterial strains and growth conditions

To study the effective constriction dynamics of the Z-ring, we used the EC448 strain of *E. coli* [courtesy of Weiss (Weiss et al., 1999)]. Its chromosome contains *ftsZ-gfp* under the control of a weakened *trc* promoter at the lambda attachment site (*attB*). In turn, the *trc* promoter is regulated by isopropyl-β-D-thiogalactoside (IPTG). While FtsZ-GFP displays a similar localization pattern as the wild type FtsZ, it does not fulfill its function in the septation process (Ma et al., 1996). At low levels of IPTG induction (40μM), it was shown that in EC448 the fraction of FtsZ-GFP of the total amount of FtsZ in the cell is between 30 and 40% and cells manifest normal growth (Thanedar and Margolin, 2004). In our experiments, FtsZ-GFP expression was induced at two IPTG concentrations: (1) weak induction at 40μM and (2) strong induction at 500μM. We found that the growth rate of strong induction cells was not affected by the addition of the IPTG and the 3D structure of the Z-ring appears to be similar in both weak and strong induction cells. Note that the induction time used in this work (1 h) is significantly shorter than in Thanedar and Margolin (2004) and Tsukanov et al. (2011) (2–3 and 1.5–2h, respectively). Our approach, namely, using high IPTG concentration and short induction time, leads to relatively uniform expression levels in the cell population (Siegele and Hu, 1997), reducing the effect of expression level variability on Z-ring images from different cells. To estimate the relative FtsZ-GFP expression level, we have compared the average fluorescence intensity within cells and found that it is only twice larger in strong induction experiments than in weak induction experiments (despite a 12.5 ratio in IPTG concentration). Therefore, in our strong induction experiments the amount of FtsZ-GFP/cell is in the same range as in the work of Thanedar and Margolin (2004).

Cells were grown at 37°C in Luria broth (LB) until OD_600_ = 0.1 in the exponential regime. At this optical density we added the IPTG to the cell medium and allowed cells to grow for another 1 h. For microscopy, the cell solution was further diluted 100 times in LB with IPTG.

### Microscopy and optical trapping

The experimental system was described in detail elsewhere (Carmon et al., 2012, 2014). Briefly, we use an inverted microscope (IX70, Olympus) with a 100X objective (UPLFLN 100XO2PH, 1.3 NA, oil immersion) and a cooled CCD (CoolSNAP ES^2^, Photometrics) to image individual *E. coli* cells. The optical trap consists of a laser beam (SDL, λ = 830 nm) that is focused onto the (*x, y*) imaging plane via the microscope objective. The trap is stiffer in the (*x, y*) plane than along the optical axis (*z* axis), such that trapped cells are aligned with their long axis in the *z* direction (vertical orientation). This allows imaging the Z-ring as a circular structure. To view cells with their long axis in the (*x, y*) plane (horizontal orientation), we use a galvanometric mirror that oscillates the trap along the *x* axis with a frequency of ~100 Hz and an amplitude equal to the cell length. This leads to an effective linear trap and to horizontal aligning of the trapped cell. Cells can be imaged in either phase contrast or fluorescence for both horizontal and vertical orientations. Our system allows switching within a few seconds between each of these four different imaging modes.

## Results

**Figure 2:**
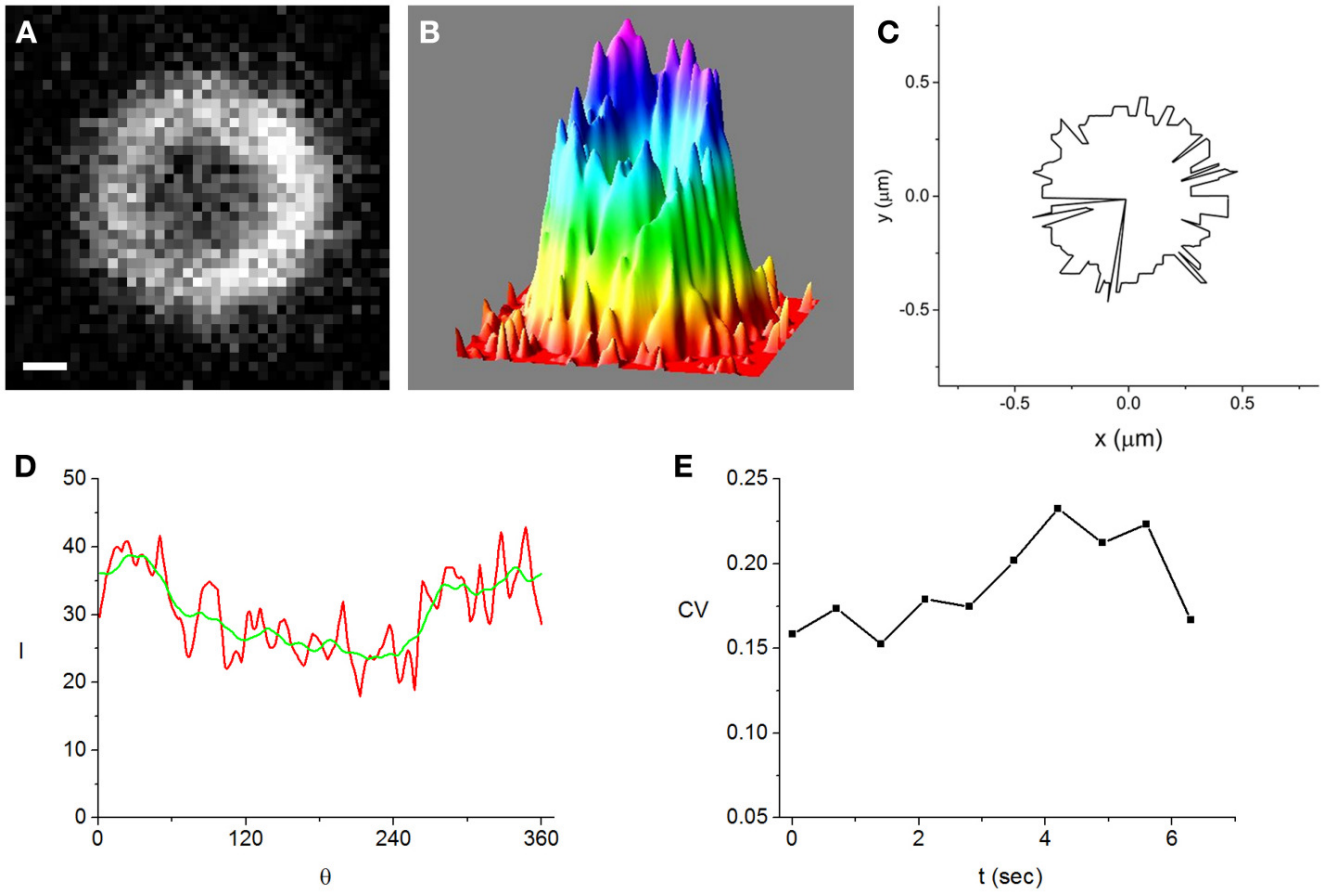
A Z-ring that is among the most inhomogeneous of the cells that we analyzed in strong induction experiments (500 μM IPTG). Bar = 0.2 μm. **(A)** fluorescence image of the ring obtained while the cell is trapped in the vertical orientation, **(B)** intensity distribution corresponding to the image shown in **(A), (C)** maximal intensity angular contour tracing the position where the radial profiles are maximal, *r*_*z*_(θ), **(D)** intensity along the maximal intensity angular contour, *I*(θ) (red). It is radially averaged over a 21 nm wide strip centered at *r*_*z*_(θ). For comparison, the angularly smoothed *I*(θ) over a range of the order of the optical resolution, ~250 nm, is also shown (green), **(E)** time-dependence of the coefficient of variation (CV) (see text). It measures the degree of inhomogeneity of the angularly smoothed *I*(θ).

Constricting cells, in which the FtsZ-GFP expression was induced at 500 μ M IPTG, were imaged in each of the four modes starting with the vertical GFP fluorescence. The latter and the horizontal phase contrast mode were used to obtain quantitative data on the Z-ring dynamics. We use the vertical GFP fluorescence images to measure the ring radius, *r*_*z*_ (see Figure 1), whenever the contrast was beyond a certain level previously shown to ensure accurate values of *r*_*z*_ (Carmon et al., 2012, 2014). The threshold contrast level was determined in calibration experiments where *r*_*z*_ is computed for an individual cell while the fluorescence intensity is gradually bleached due to repeated exposures. We find the best fitting circle to the ring image and compute radial intensity profiles along 360 rays that radiate from the center of this circle at 1° intervals (Carmon et al., 2012, 2014). Next, we average the radial profiles and define *r*_*z*_ as the distance from the center to the point where the average radial profile is maximal. Unlike in 3D-PALM imaging of the Z-ring where it was shown that most rings are incomplete (Holden et al., 2014; Coltharp et al., 2016), we obtain ring images that are remarkably homogeneous along their circumference (Figures 2A–D and Figures S2A–D). This difference in the appearance of the Z-ring can only partially be due to the limited resolution of our system. Diffraction will not obscure gaps in the circumference of the Z-ring with an angular extent of more than about 30°, while most of the Z-ring gaps in Holden et al. (2014) and Coltharp et al. (2016) are much larger. Instead, the large gaps found in the 3D-PALM studies are due to the low level of FtsZ in the slow growing cells. The ring homogeneity that we find in our experiments is consistent with the results from 3D-SIM (Rowlett and Margolin, 2014), where Z-rings of fast growing *E. coli* were found to be continuous aside from holes typically smaller than the diffraction limit. Accordingly, in our images such holes represent an extremely rare occurrence found only in the most inhomogeneous ring images that were recorded (Figures 2A–D). We note that Rowlett et al. (Rowlett and Margolin, 2014) used either FtsZ-GFP at weak induction (30 μ M IPTG) or immunofluorescence to image Z-rings, obtaining similar, relatively homogeneous distributions of FtsZ along the ring circumference with no gaps larger than ~250 nm. This suggests that, in their experiments, the absence of large gaps in the Z-ring is not due to the additional FtsZ-GFP in the cell, which seems to have no visible effect on the FtsZ distribution in the ring.

To characterize the inhomogeneity in the ring images we use the angular intensity profiles averaged over a strip of 21 nm centered at *r*_*z*_, *I*(θ) (Figure 2D and Figures S1D, S2D, S3). We find that the angular profiles are noisy but do not vary much on scales larger than the optical resolution, ~250 nm. Using the coefficient of variation (CV ≡ standard deviation/average) of the *I*(θ) smoothed over an angular range corresponding to the optical resolution to quantify the ring inhomogeneity, we note that it is similar in the most homogeneous and most inhomogeneous rings. Moreover, the CV's are roughly constant on a 10 s time scale (Figure 2E and Figure S2E) and in the same range for weak induction cells (Figure S1E). Although the fluctuations in the time dependence of the CV for weak induction cells (Figure S1E) appear to be larger than those of strong induction cells (Figure 2E), the difference between them is not statistically significant (see Supplementary Material).

Although the degree of inhomogeneity in the angular profiles is approximately constant on short time scales and at different levels of induction, several factors lead to significant errors in the measured values of *r*_*z*_. Due to the angular inhomogeneity of the ring in an individual cell, the maximal intensity in the radial profiles corresponding to different angles is located at slightly different radial distances, *r*_*z*_(θ) (Figure 2C and Figure S2C). Since *r*_*z*_ ≈ < *r*_*z*_(θ) >, the error of *r*_*z*_, Δ*r*_*z*_, contains a contribution due to the deviations of *r*_*z*_(θ) from its average. Another contribution to Δ*r*_*z*_ is due to the Brownian motion of the trapped cell. In the vertical orientation, the peak to peak amplitude of these fluctuations is about 100 nm. Although this length scale is below optical resolution, ~250 nm, these fluctuations will lead to an additional smearing of the ring image. Moreover, the Brownian cell motion affects the ring image by varying its distance from the focal plane. To reduce Δ*r*_*z*_, we average the measured values of *r*_*z*_ over multiple frames (exposure time = 0.5 s, time between frames = 0.2 s). Out of the 50 frame movies recorded for each cell, we select only the *N* frames for which the ring image satisfies our minimal contrast requirement. For the time averaged *r*_*z*_, the error is defined as the standard deviation divided by $\sqrt{N}$. For most cells, this error is of the order of only a few nm and its average over all cells is 4.6 nm.

We have used the procedure described above to measure *r*_*z*_ for 25 cells that displayed varying depths of constriction. To compare the geometry of the Z-ring with that of the septum at different stages of cell division, we also measured the constriction radius, *r*, and the cell radius, *R*, for each of these cells (Figure 1). To this end, we use the horizontal phase contrast imaging mode and obtain cell contours with sub-pixel accuracy. Our approach to computing such contours from phase contrast images was described in Reshes et al. (2008a). We stain the cytoplasmic membrane with FM4-64 to obtain the average value of the normalized phase contrast intensity that corresponds to the edge of the cell. For this calibration, we analyzed seven trapped cells and obtained a phase contrast threshold value. On a rescaled intensity axis where the background is set to unity and the interior of the cell to zero, we find that the cell edge corresponds to an intensity value of 0.56. Using 2D linear interpolation to obtain intensity values at positions in between pixel centers allows determining the cell contour with an accuracy of ~30 nm. The value of the constriction radius, *r*, is best estimated as half the shortest distance between two contour points on opposite sides of the cell not too far from midcell (see Figure 1). Moreover, we approximate the cell radius, *R*, as half the average distance between the opposite cell sides away from the constriction region and from the cell caps (Figure 1). Similar to the case of *r*_*z*_, here as well, the measurement errors of *r* and *R*, Δ*r*, and Δ*R*, although much smaller than the optical resolution, are nevertheless too large to obtain the behavior of *r*_*z*_(*r*) with sufficient accuracy. Specifically, 25 nm < Δ*r* < 53 nm and 7 nm < Δ*R* < 19 nm. As before, we use time averaging to reduce Δ*r* and Δ*R*. We record a 50 frames movie in the horizontal phase contrast mode (exposure time = 0.02 s, time between frames = 0.2 s) instead of a single image and average the values of *r* and *R* over all frames. The errors of these averages are all less than 3 nm.

**Figure 3:**
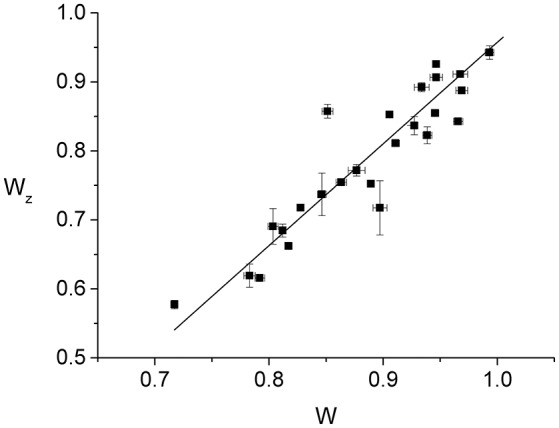
Dependence of the normalized Z-ring radius, *W*_*z*_ ≡ *r*_*z*_/*R*, on the normalized radius of the constriction, *W* ≡ *r*/*R*. Each of the data points (squares with error bars) represents the measurement from an individual cell. The error bars are due to the fluctuations in the values of either *W*_*z*_ or *W* between the frames used for the time averaging. While for *W* we averaged over 50 consecutive frames, in the case of *W*_*z*_ the number of frames entering the average varied between 4 and 40 as only images with sufficient contrast were included. The 25 cells of the data set were measured in 9 different strong induction experiments (500 μM IPTG). The corresponding best linear fit to the data is also shown (line).

Since individual cells have slightly different radii, it is expected that normalizing the Z-ring radius, *r*_*z*_, and the constriction radius, *r*, by the cell radius, *R*, will reduce the effect of the population variability. Accordingly, we find that the normalized Z-ring radius, *W*_*z*_ ≡ *r*_*z*_/*R*, appears to vary linearly as a function of the normalized constriction width, *W* ≡ *r*/*R*, up to fluctuations that are probably due to population variability (Figure 3). The best linear fit to the data, *W*_*z*_ = *aW* + *b*, corresponds to *a* = 1.47 ± 0.02 and *b* = −0.52 ± 0.01, indicating that the Z-ring constricts faster than the cell septum (see Supplementary Material). Since the outer edge of the Z-ring is attached to the cytoplasmic membrane and its radius, *r*_*z*_, the position of the maximal intensity of the radial profile, corresponds to the middle of the ring's radial width, the behavior of *W*_*z*_(*W*) suggests that, for fast growing cells, the radial width of the ring grows as the constriction is progressing. While this behavior is different from the approximately constant radial width found in slow growing cells (Coltharp et al., 2016) (see Figure S6), it represents an analogous trend to the significantly more homogeneous Z-rings than those of Coltharp et al. (2016). We expect that both these differences between our results and those of Coltharp et al. (2016) are due to the larger amount of FtsZ available in fast growing cells.

To obtain the constriction dynamics of the Z-ring, we establish the effective division time for each individual cell using the corresponding values of *r* and *R*. We have previously shown that the normalized constriction width of individual cells, *W*, follows a simple dynamics

(1) $$\begin{array}{r} W\left(t\right)=\sqrt{1-{\left(\frac{t-\tau_{c}}{\tau_{g}-\tau_{c}}\right)}^{2}}\equiv \sqrt{1-{\left(t^{\prime }\right)}^{2}} \end{array}$$

where τ_*c*_ is the time at the onset of constriction, τ_*g*_ is the generation time and $t^{\prime }\equiv \frac{t-\tau_{c}}{\tau_{g}-\tau_{c}}$ is the effective division time. The time interval between τ_*c*_ and τ_*g*_, τ_*g*_ − τ_*c*_, corresponds to the T-period of the cell cycle (Den Blaauwen et al., 1999). Equation (1) was derived in Reshes et al. (2008a) using the following four assumptions (see Figure 1): (1) the cell is shaped as a cylinder with two hemispherical caps, (2) its septum consists of two incomplete hemispheres; as division proceeds these grow to form the new caps, (3) the septal area grows at a constant rate, and (4) the cell radius, *R*, is constant in time. Its prediction was compared to measurements from individual fast growing *E. coli* cells and found to be in excellent agreement with experiment (Reshes et al., 2008a,b). Inverting Equation (1) we obtain *t*′(*r*/*R*) and the error of *t*′, Δ*t*′, as a function of *r*, *R*, Δ*r*, and Δ*R*.

**Figure 4:**
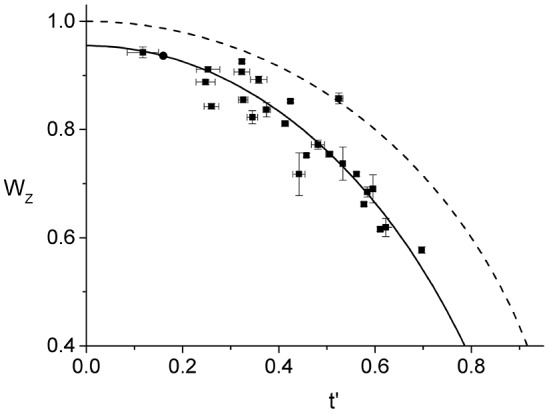
Effective dynamics of the Z-ring constriction, $W_{z}\left(t^{\prime }\right)$ (squares with error bars). We use the same data set as in Figure 3, only here the normalized radius of the Z-ring is plotted as a function of the effective division time. The prediction of Equation (1) (dashed line) together with the curve corresponding to the linear fit in Figure 3 (full line) are also shown.

In Figure 4, we show the behavior of $W_{z}\left(t^{\prime }\right)$ for all the 25 cells that we have analyzed. As one would expect, using the parameters from the best linear fit of Figure 3 to obtain the effective time, *t*′, for each individual ring, leads to a $W_{z}\left(t^{\prime }\right)$ curve that is in good agreement with the corresponding data. In contrast, assuming that the ring radius constricts at the same rate as the cell septum provides a relatively poor description of the observed behavior (see Figure S6). Comparing between $W_{z}\left(t^{\prime }\right)$ and *W*(*t*), indicates that the ring radius constricts at a faster rate than the cell septum and, correspondingly, the radial width of the ring grows as a function of the effective division time.

Note that the data for $W_{z}\left(t^{\prime }\right)$ only represents cells with rings that are not too small, *r*_*z*_ > 0.6*R*, and not too late during septation, *t*′ < 0.7. The restricted range of the data is due to two inherent limitations of our experimental approach. First, the cell edge calibration is performed on unconstricted cells and its validity for the case of the septal geometry holds only as long as the contributions from the two incomplete caps are sufficiently well-separated. In previous work we have determined that our edge calibration fails whenever the septation is deeper than 55%, *W* < 0.55 (Reshes et al., 2008a). Second, we found that the vertical trapping stability of cells that exceed a certain length is gradually deteriorating. For such cells fluctuations become too large and the corresponding ring images cannot be used for quantitative analysis due to the additional blur.

A necessary ingredient in our experimental approach is using time averaging to reduce the measurement error. Prior to averaging, errors in the effective time, *t*′, were as large as 45%, and in the normalized ring radius, *W*_*z*_, up to 7%, making the data practically useless. In fact, the larger errors bars on several of the *W*_*z*_ data of Figures 3, 4 are due to the small number of frames used for the averaging, e.g., at *W* = 0.9 (corresponding to *t*′ = 0.44) only four frames were used since for later ones bleaching reduced contrast below the required value. The averaging procedure is reducing both the random errors, like those due to Brownian motion and imaging, and those due to the angular inhomogeneity of the Z-ring intensity distribution. The error due to inhomogeneity is reduced since the angular distribution of FtsZ in the ring varies on a time scale shorter than that of the averaging (see Supplementary Material and Figure S3).

## Discussion

We have used Optical Tweezers and subpixel image analysis to monitor the changes in the geometry of the Z-ring during division in *E. coli*. The optical trap allows orienting a cell either horizontally or vertically and fast switching between the two alignments. Previously, we employed this system to measure the radial width of the Z-ring in cells that had not yet started to septate (Carmon et al., 2012, 2014). Here, our Optical Tweezers assisted imaging approach is used to monitor the constriction dynamics of the Z-ring. Specifically, we use the horizontal view to determine the size of the constriction. It allows establishing the effective time along the division process in a particular cell. In addition, the vertical view of the Z-ring appears as a circular structure that can be quantitatively analyzed to obtain the radius of the ring, *r*_*z*_. Combining the information from the vertical fluorescence and horizontal phase contrast imaging modes, our data indicates that the Z-ring constricts faster than the cell envelope suggesting that the radial width of the ring grows during cell division. Moreover, we find that the Z-rings are significantly more homogeneous than those of slow growing cells. Our results together with those of Coltharp et al. (2016) suggest that both the geometry and the constriction dynamics of the Z-ring are different between fast and slow growing cells.

A possible mechanism that leads to the different behaviors of the Z-ring in fast and slow growing cells assumes that there is a limited amount of FtsZ protein available in slow growing cells. In contrast, in fast growing cells the amount of FtsZ is sufficient to build a Z-ring that appears roughly homogeneous on the scale of the diffraction limited optical resolution, ~250 nm. This assumption is consistent with earlier results showing that the number of FtsZ proteins/cell is much larger in fast growing than in slow growing cells (Link et al., 1997; Lu et al., 1998; Rueda et al., 2003; Ishihama et al., 2008; Vendeville et al., 2011). Moreover, a recent study found that the Z-rings become gradually more homogeneous as the expression level of FtsZ is enhanced providing further support for this scenario (Lyu et al., 2016).

Although 3D-PALM is certainly a more advanced microscopy technique than our Optical Tweezers assisted imaging approach, for the particular purpose of measuring the radius of the bacterial Z-ring the two methods provide results of comparable accuracy and are complementary with respect to the conditions that optimize their performance. First, we bypass the optical resolution limit using pixel interpolation together with space and time averaging to obtain the radius of the Z-ring for individual cells, *r*_*z*_, with an average error of only 4.6 nm. This accuracy is comparable to that obtained using iPALM (Coltharp et al., 2016). However, the high accuracy of our approach is partly due to the relatively homogeneous Z-rings that were measured in this study and will deteriorate as the ring under investigation becomes more inhomogeneous as in Coltharp et al. (2016). Second, the error of the measured Z-ring radius of individual cells is not specified in Holden et al. (2014) and Coltharp et al. (2016). Since most of the quantitative analysis is performed on the population averages, the errors of the different Z-ring characteristics are obtained from the variations between cells. This approach does not distinguish between measurement errors and population variability. In contrast, in our analysis of the Z-ring constriction dynamics we use the Z-ring radii from individual cells together with their corresponding measurement errors (Figures 3, 4), allowing to isolate the effect of population variability. Third, Coltharp et al. (2016) use fixed cells to image the Z-ring with 3D-PALM. In their experiments, cells were fixed with 4% formaldehyde, potentially causing various artifacts. Such a high concentration of formaldehyde could shift the equilibrium in dynamic protein complexes, as is the case for most other cross-linkers. Moreover, the small size of formaldehyde limits its spatial and temporal cross-linking ability (Kiernan, 2000), leading to a wide variability in the extent it is immobilizing different proteins (Tanaka et al., 2010). In contrast, we study live cells therefore avoiding artifacts due to fixation. This also allows us to monitor the short time dynamics of the FtsZ distribution along the circumference of the ring (Figure 2E, Figures S1E, S2E, S3).

Our main assumption leading to the conclusion that the ring constricts faster than the cell septum is that the maximal intensity of the average radial profile corresponds to the radius of the Z-ring. It ignores the potential contribution of the FtsZ-GFP that is distributed in the cell cytoplasm, about 70% of the total amount of FtsZ-GFP in the cell (Anderson et al., 2004; Geissler et al., 2007). However, it was shown in previous work that most of this FtsZ-GFP is outside the depth of field and accordingly has a negligible contribution to the value of *r*_*z*_ (Carmon et al., 2014). To this end, we have simulated the image of the Z-ring with and without the FtsZ-GFP in the cytoplasm using the appropriate 3D point spread function for our microscope. Nevertheless, the spread of the *W*_*z*_(*W*) and $W_{z}\left(t^{\prime }\right)$ data (Figures 3, 4, respectively) could be partially due to this effect and the variability in the partition of FtsZ-GFP between the ring and the cytoplasm between the different cells.

To obtain the effective division time, *t*′, we also had to make the four geometrical assumptions leading to Equation (1). While the prediction of Equation (1) was extensively verified for fast growing *E. coli* cells (Reshes et al., 2008a,b), it apparently contradicts the behavior observed by Coltharp et al. for slow growing cells (Coltharp et al., 2016). They found that the septum constricts at an almost constant rate, such that using a generalized version of Equation (1)

(2) $$W(t\prime )={\left[1-{\left(t\prime \right)}^{\alpha }\right]}^{1{╱}_{\alpha }\;}$$

they obtain α = 1.3 ± 0.1. However, it is possible that, in a regime where cells are growing at a slow rate, their septum will tend to display a more linear constriction dynamics. That is, we suggest that the different septation rates correspond to the different growth rate regimes, such that, for fast growing cells, α ≃ 2 (Reshes et al., 2008a,b), while for slow growing cells, α ≃ 1.3 (Coltharp et al., 2016). The fact that one of the wild type strains analyzed in Coltharp et al. (2016), MC4100, with an intermediate generation time, τ_*g*_ = 114 ± 3 min, was found to constrict with α = 1.9±0.2, consistent with the prediction of Equation (1), lends some support to this view (although the BW25113 strain when grown in rich medium, τ_*g*_ = 94 ± 2 min, constricted according to Equation 2 with α = 1.3 ± 0.1). Moreover, the two constriction rate regimes scenario is compatible with the other differences between fast and slow growing *E. coli* cells, namely: (1) the growth of the cylindrical cell region stops at the onset of septation in slow growing cells while it is only slightly reduced in fast growing cells, (2) the Z-ring is much more homogeneous along its circumference in fast growing than in slow growing cells and (3) the radial width of the Z-ring grows during septation in fast growing cells while it remains approximately constant in slow growing cells.

Despite recent progress understanding the role of the Z-ring in cytokinesis (Bisson-Filho et al., 2017; Yang et al., 2017), the mechanism that enables the constriction of the ring is still being debated (Ghosh and Sain, 2008; Allard and Cytrynbaum, 2009; Erickson, 2009). Recently, Coltharp et al. (2016) have shown that several properties of the Z-ring, namely, its density, the GTPase activity of FtsZ and the timing of its assembly and disassembly, had practically no effect on the rate of constriction. Moreover, the significant difference in the homogeneity of Z-rings in normally dividing cells between the fast and slow growth regimes, including incomplete rings with an angular range of less than 180°, also indicates that Z-ring constriction is not due to force exerted by the ring itself. Specifically, a less than 180° arch-like Z-ring cannot constrict toward the cell axis without guidance from the circularly symmetric cell wall, a necessary pathway to obtaining a circularly symmetric septum at midcell. These findings suggest that Z-ring constriction is led by the assembly of the septating peptidoglycan cell wall.

## Author contributions

PK performed the experiments and analyzed the data. AY assisted with the experiments and the optical alignment of the Optical Tweezers. MF designed the research. IF and MF supervised the experiments and data analysis and wrote the paper.

### Conflict of interest statement

The authors declare that the research was conducted in the absence of any commercial or financial relationships that could be construed as a potential conflict of interest.
