# Supplementary material for: Z-ring Structure and Constriction Dynamics in E. coli
Source: Front Microbiol. 2017 Sep 11;8:1670. doi: 10.3389/fmicb.2017.01670 (PMC5603902; doi:10.3389/fmicb.2017.01670)
Supplement: Supplementary file 1 [file Presentation1.PDF]

Supplementay Material

Z-ring structure and constriction dynamics in *E. Coli*

P. Kumar, A. Yadav, I. Fishov and M. Feingold

*** Correspondence:** M. Feingold: mario@exchange.bgu.ac.il

1 Supplementary Data

1.1 Z-ring in weak induction cells

Most of the Z-ring images that we obtain for cells induced with low levels of IPTG (40 μM) had low contrast and are hard to analyze quantitatively. In Fig. S1 we show a typical example from the few weak induction cells that satisfy our minimal contrast requirement. Although it displays a small gap (Fig. S1c), its image is not significantly more inhomogeneous than that of cells from strong induction experiments. Moreover, the ring images of the later frames are more uniform than that of the first frame (Figs. S1a-d) as indicated by the time dependence of the CV (Fig. S1e).

For a quantitative comparison of the inhomogeneity between the cells of Figs. 2 and S1 we may use the time dependence of the CV in Figs. 2e and S1e. We find that the strong induction cell has an average CV of 0.19 with a standard deviation of 0.039. The corresponding values for the weak induction cell of Fig. S1 are 0.13 and 0.028, respectively. Therefore, the weak induction cell of Fig. S1 is on average more homogeneous than one the most inhomogeneous strong induction cells, namely, that of Fig. 2, since the difference between their average CV's, 0.055, is significantly larger than the errors of the average CV's, 0.012 and 0.009, respectively. This suggests that the relative circumferential homogeneity of the Z-ring in our experiments is not due to using a high concentration of IPTG.

The time dependence of the CV for the strong induction cell of Fig. 2 also has a lower variability relative to that of the weak induction cell of Fig. S1, as its standard deviation is 0.028, while that of the weak induction cell is 0.039 (Figs. 2e and S1e). However, the errors of these standard deviations, 0.006 and 0.009, respectively, add up to more than the difference between their values, $0.015 > 0.011$, such that the difference between the CV(t) variability of these two cells is not statistically significant.

1.2 Short time dynamics of the Z-ring inhomogeneity

While the Z-ring shown in Fig. 2 is one of the most inhomogeneous among the cells that were imaged in our experiments, at the opposite extreme, in Fig. S2 we show one of the most homogeneous rings. The extent of variation in the intensity along the ring circumference is not much lower than that in the inhomogeneous ring of Fig. 2. Moreover, we find that, for both

homogeneous and inhomogeneous rings, $I(\theta)$ displays rapid time dependence on a time scale faster than the rate of our imaging (Fig. S3).

Specifically, we monitor the dynamics of the Z-ring angular intensity profile, $I(\theta)$, at 0.7 sec time intervals (Fig. S3) and find that it varies significantly between consecutive frames. In other words, the FtsZ angular distribution corresponding to a particular frame is mostly independent of the distributions of the other frames. Consequently, the time averaging of r_z not only reduces the effect of Brownian fluctuations, but also lowers the error due to the dynamic inhomogeneity in the distribution of the ring material.

1.3 Z-ring radius is not affected by the optical trap

Laser exposure is known to lead to photodamage in live cells. For the case of *E. coli*, it was shown that below total exposure of about 0.36 J, cells display normal growth and division behavior (1). In this range, there was no difference in the cytokinesis between individual cells that were exposed to the laser and those that were not. In our experiment, the laser power at the exit from the microscope objective is 37 mW, corresponding to a time limit of about 10 sec before the onset of photodamage. However, it was shown that photodamage is about two times lower at the wave length of our laser, 830 nm, than at that of the Nd:YAG laser (λ = 1064 nm) used by Ayano *et al.* (1, 2). We may therefore expect that in our experiments trapped cells would not experience any photodamage within a time window of about 20 sec.

On one hand, to acquire a large enough number of frames for the time averaging, we record the Z-ring fluorescence in the vertical mode for up to 30 sec. On the other hand, we find that on this time scale the photodamage has negligible influence on the value of the Z-ring radius, r_z. Comparing the values of r_z obtained from the first 10 sec of the experiment (frames 1 to 15), $r_{z,1}$, with those from the next 10 sec (frames 16 to 30), $r_{z,2}$, (Fig. S4), we find that the values of $r_{z,1}$ and $r_{z,2}$ are equal within the experimental error.

The photodamage may also affect our measurements of the cell morphology from the horizontal phase contrast images. Specifically, the values of the constriction radius that are measured up to about 60 sec from the start of the trapping may have been affected by the cell being exposed to the laser beam. To verify this possibility we measured the values of the constriction radius, r, for the first 25 frames, r_1, and for the next 25 frames, r_2. As in the case of r_z, here as well, we find that the values of r_1 and r_2 are equal within the experimental error. This suggests that, on this timescale, the effect of photodamage on the value of the constriction radius is negligible. We find that this statement also holds for the case of the cell radius, R.

1.4 Dependence of the normalized Z-ring radius, W_z, on the normalized septal radius, W

The behavior of the $W_z(W)$ data shown in Fig. 3 is well approximated by the corresponding linear fit, $W_z = a\,W + b$, where $a = 1.47 \pm 0.02$ and $b = -0.52 \pm 0.01$. These values of a and b were obtained using standard least-squares fitting where the contribution of each data point is

weighted by the corresponding error of the normalized Z-ring radius, ΔW_z. Specifically, we minimize the $\chi^2(\tilde{a},\tilde{b})$ function with respect to the $\tilde{a}$ and $\tilde{b}$ parameters, where

$$\chi^2(\tilde{a},\tilde{b}) = \sum_{i=1}^{N} \frac{(W_{z,i} - \tilde{a}W_i - \tilde{b})^2}{(\Delta W_{z,i})^2} \qquad (1)$$

and the sum runs over the *N* data points. This ensures that data points with large errors in W_z, e.g. (W, W_z) = (0.9, 0.7), will have a smaller weight in determining the value of the best fitting line than points with small errors, e.g. (W, W_z) = (0.72, 0.58).

Alternatively, the fit in Fig. 3 can be done using a generalization of Eq. (1) that also includes the effect of the errors in the normalized septal radius, ΔW. The statistical theory behind such generalization is significantly more involved than Eq. (1) (3). In particular, the results of the fit depend on the correlation coefficient, *r*, between ΔW and ΔW_z. Assuming that ΔW and ΔW_z are uncorrelated, $r = 0$, leads to a linear fit that is only slightly different from that of Fig. 3, namely, $a = 1.53 \pm 0.02$ and $b = -0.56 \pm 0.02$, such that the two lines are practically overlapping. Moreover, using the value of r computed from the actual data, $r = 0.27$, leads to values of a and *b* that only differ from the $r = 0$ in the third digit after the decimal point.

For completeness, we also present the parameters of the linear fit to the $W_z(W)$ data for the case when all measurements are given equal weights ignoring the variation in the corresponding errors. Although in this case we obtain a lower value for the slope, $a = 1.37 \pm 0.03$ and $b = -0.42 \pm 0.02$, it is nevertheless significantly larger than 1.

1.5 FM4-64 membrane staining and its effect on the behavior of $W_z(W)$ and $W_z(t')$

Our measurements of the cell radius, R, and of the constriction radius, r, rely on the calibration of the phase contrast intensity that corresponds to the position of the cytoplasmic membrane. For this calibration we use the FM4-64 fluorescent stain assuming that it is localized in the cytoplasmic membrane (4). However, other studies have found evidence that FM4-64 stains the outer rather than the inner membrane (5, 6). Although this is an important controversy it only has a small effect on our results (Fig. S5). Using the data of Szwedziak *et al* (Fig. 1F of Ref. (7)) we can estimate the distance between the inner and outer membranes in *E. coli* at about 16 nm. Therefore, the effect on our data of staining the outer membrane instead of the cytoplasmic membrane is that the measured values of R and r are larger by ~16 nm than their true value. In Fig. S5 we illustrate the effect of subtracting the 16 nm inter-membrane distance from the R and r values for all the cells in our data set. As one would expect, while the intercept of the best linear fit to the adjusted $W_z(W)$ data, $W_z = a_1 W + b_1$, is slightly lower than that of Fig. 3, $b_1 = -0.49 \pm 0.01$, the corresponding slope remains practically unchanged, $a_1 = 1.48 \pm 0.02$ (Fig. S5a). Here, the best fit was obtained using the χ^2-function of Eq. (1), same as in Fig. 3. Moreover, the quality of the fit is the same as that in Fig. 3, for both $R^2 = 0.87$ (R^2 refers to the coefficient of determination, the standard measure for the quality of the fit).

The only consequence of assuming that the FM4-64 stains the outer rather than the inner membrane is best illustrated in the effective time dependence of the normalized ring radius, $W_z(t')$ (Fig. S5b). It shows that the reduced value of the cell radius leads to a significantly thinner Z-ring at the onset of constriction, $t'=0$. As discussed in relation to Fig. 3, the width of the Z-ring, Δ, is twice the difference between the constriction radius, r, and the radius of the Z-ring, r_z, $\Delta = 2(r - r_z)$. Using the data of Fig. S5, this leads to $\Delta(t'=0) = 10 \pm 22$ nm for the width of the unconstricted Z-ring, while $\Delta(t'=0) = 42 \pm 22$ nm for the data in Figs. 3 and 4. As one would expect, the difference between these two values of $\Delta(t'=0)$ is precisely 32 nm, namely, twice the inter-membrane distance that we assumed.

On one hand, the width of the unconstricted Z-ring obtained assuming that FM4-64 stains the inner membrane is consistent with the results from the various optical microscopy studies (8-11). On the other hand, the $\Delta(t'=0) = 10 \pm 22$ nm Z-ring width is consistent with the electron microscopy measurements suggesting that the FtsZ protofilaments are organized in a single layer adjacent to the cytoplasmic membrane (7, 12). Therefore, two apparently unrelated controversies, FM4-64 inner or outer membrane staining and the width of the unconstricted Z-ring are seemingly linked. While we cannot provide a definite answer to neither of these open questions, our finding that the width of the Z-ring increases during constriction suggests that it has a multilayered architecture, consistent with the wide Z-ring scenario in unconstricted cells.

1.6 A Z-ring model assuming constant radial width does not provide the best description of the data

Coltharp *et al.* have shown that for slow growing *E. coli* cells the radial width of the Z-ring is approximately constant during septation up to the point where it starts to collapse (10). Therefore, according to their data the Z-ring constricts at a similar rate as the cell envelope. This behavior is different from what we find for fast growing cells. In Fig. S6 we compare our data for $W_z(W)$ and $W_z(t')$ with the predictions of a Z-ring model assuming constant radial width and show that it does not describe the data nearly as well as the growing radial width model presented in Figs. 3 and 4.

1. Ayano, S., Y. Wakamoto, S. Yamashita, and K. Yasuda. 2006. Quantitative measurement of damage caused by 1064-nm wavelength optical trapping of *Escherichia coli* cells using on-chip single cell cultivation system. Biochem Biophys Res Commun 350:678-684.
2. Neuman, K. C., E. H. Chadd, G. F. Liou, K. Bergman, and S. M. Block. 1999. Characterization of photodamage to *Escherichia coli* in optical traps. Biophys J 77:2856-2863.
3. York, D., M. N. Evensen, M. L. Martinez, and J. D. Delgado. 2004. Unified equations for the slope, intercept, and standard errors of the best straight line. Am J Phys 72:367-375.

4. Fishov, I., and C. L. Woldringh. 1999. Visualization of membrane domains in Escherichia coli. Mol Microbiol 32:1166-1172.

5. Lewenza, S., D. Vidal-Ingigliardi, and A. P. Pugsley. 2006. Direct visualization of red fluorescent lipoproteins indicates conservation of the membrane sorting rules in the family Enterobacteriaceae. J Bacteriol 188:3516-3524.

6. Zupan, J. R., T. A. Cameron, J. Anderson-Furgeson, and P. C. Zambryski. 2013. Dynamic FtsA and FtsZ localization and outer membrane alterations during polar growth and cell division in Agrobacterium tumefaciens. Proc Natl Acad Sci U S A 110:9060-9065.

7. Szwedziak, P., Q. Wang, T. A. Bharat, M. Tsim, and J. Lowe. 2014. Architecture of the ring formed by the tubulin homologue FtsZ in bacterial cell division. eLife 3:e04601.

8. Biteen, J. S., E. D. Goley, L. Shapiro, and W. E. Moerner. 2012. Three-dimensional super-resolution imaging of the midplane protein FtsZ in live *Caulobacter crescentus* cells using astigmatism. Chemphyschem 13:1007-1012.

9. Carmon, G., P. Kumar, and M. Feingold. 2014. Optical tweezers assisted imaging of the Z-ring in *Escherichia coli*: measuring its radial width. New Journal of Physics 16.

10. Coltharp, C., J. Buss, T. M. Plumer, and J. Xiao. 2016. Defining the rate-limiting processes of bacterial cytokinesis. Proc Natl Acad Sci U S A 113:E1044-1053.

11. Holden, S. J., T. Pengo, K. L. Meibom, C. Fernandez Fernandez, J. Collier, and S. Manley. 2014. High throughput 3D super-resolution microscopy reveals *Caulobacter crescentus in vivo* Z-ring organization. Proc Natl Acad Sci U S A 111:4566-4571.

12. Li, Z., M. J. Trimble, Y. V. Brun, and G. J. Jensen. 2007. The structure of FtsZ filaments in vivo suggests a force-generating role in cell division. The EMBO journal 26:4694-4708.

2 Supplementary Figures and Tables

2.1 Supplementary Figures

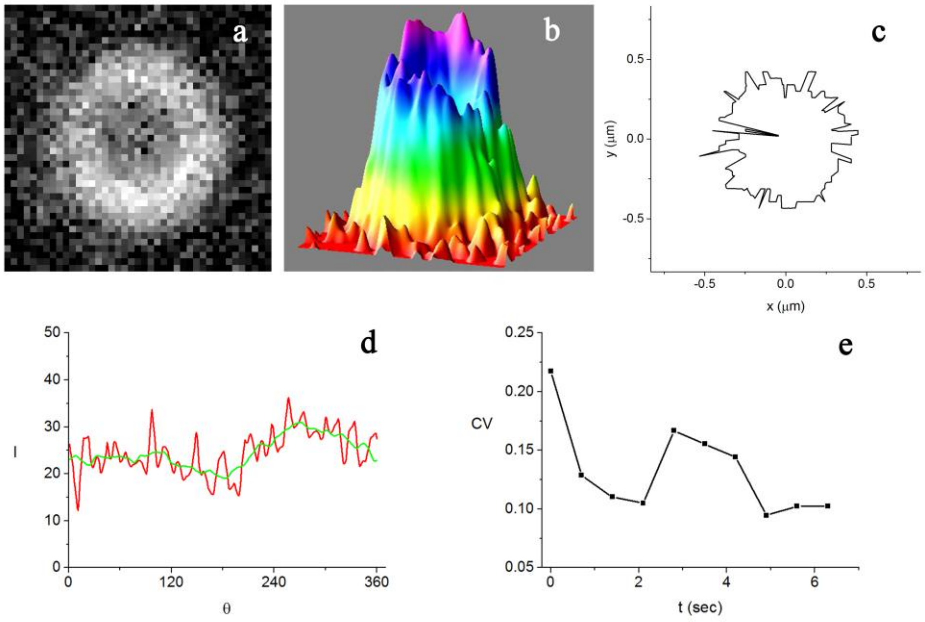

Fig. S1 – Same as in Fig. 2, only here we show the Z-ring of a cell from a weak induction experiment.

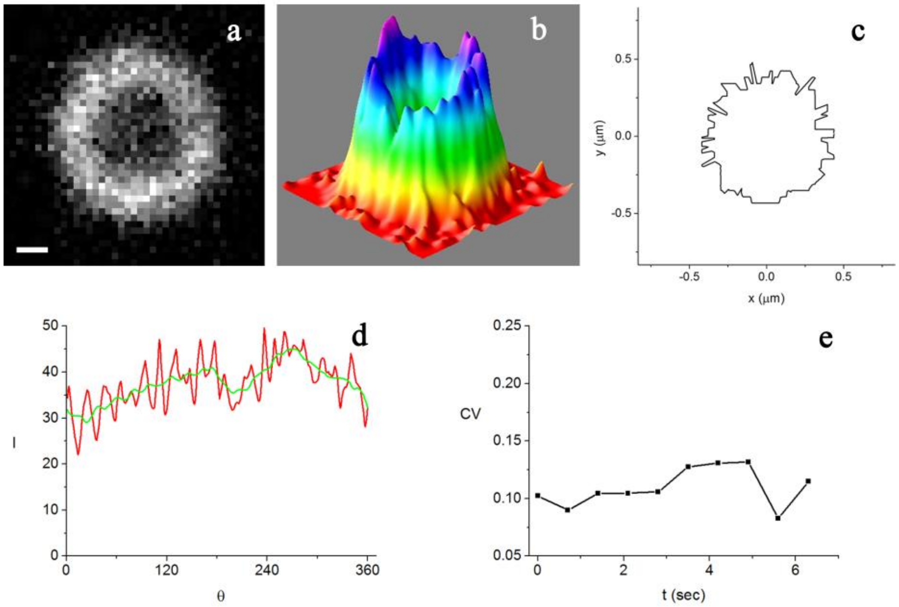

Fig. S2 – Same as in Fig. 2, only here we show one of the most homogeneous Z-rings encountered in our imaging experiments. The corresponding $I(\theta)$ plots associated with the individual time points in e) are shown in Fig. S3.

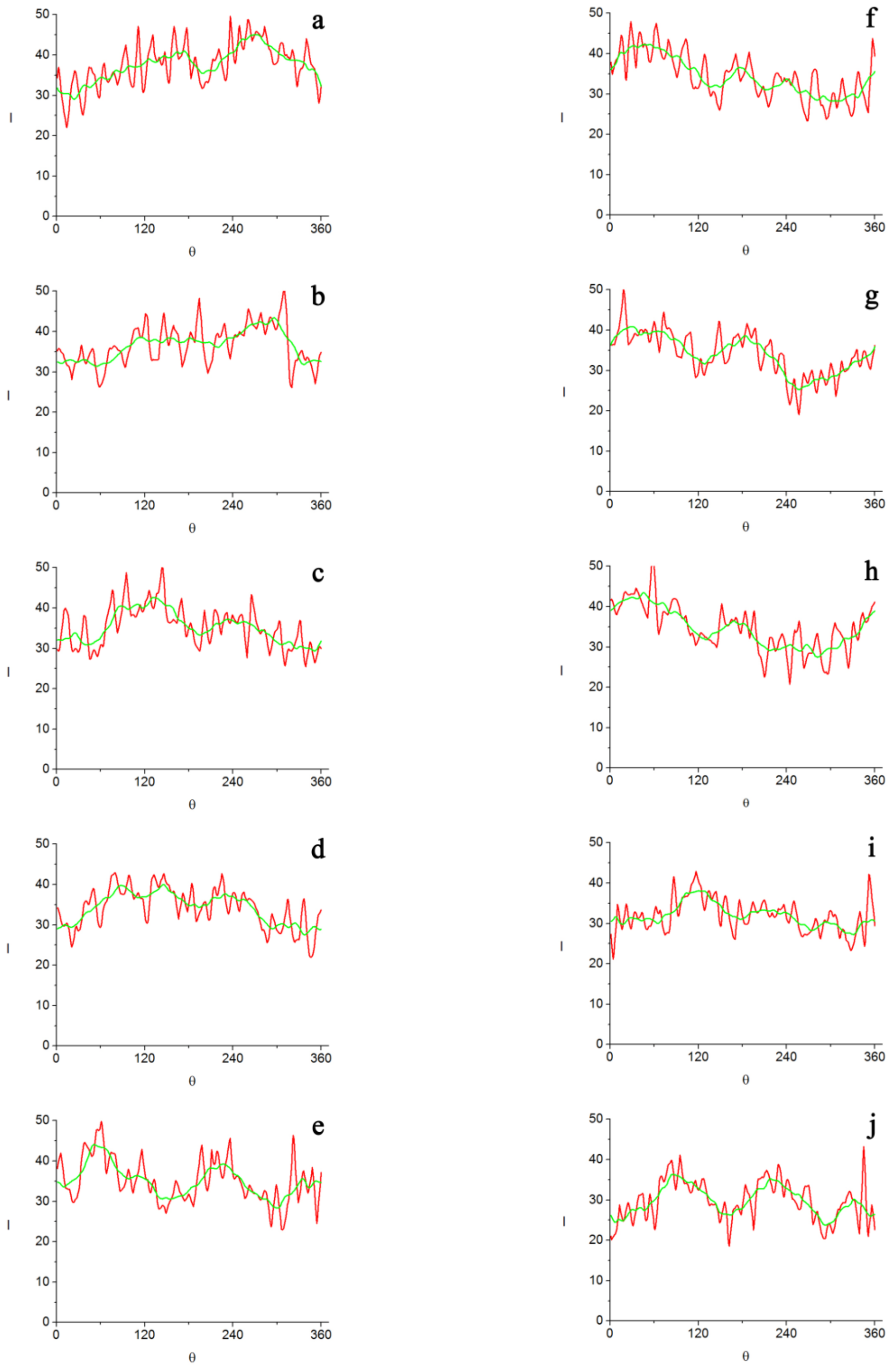

Fig. S3 – Same as in Fig. S2d shown at different times (consecutive frames) . a) t = 0.25 sec (same as Fig. S2d), b) $t = 0.95$ sec, c) $t = 1.65$ sec, d) $t = 2.35$ sec, e) $t = 3.05$ sec, f) $t = 3.75$ sec, g) $t = 4.45$ sec, h) $t = 5.15$ sec, i) $t = 5.85$ sec, and j) $t = 6.55$ sec.

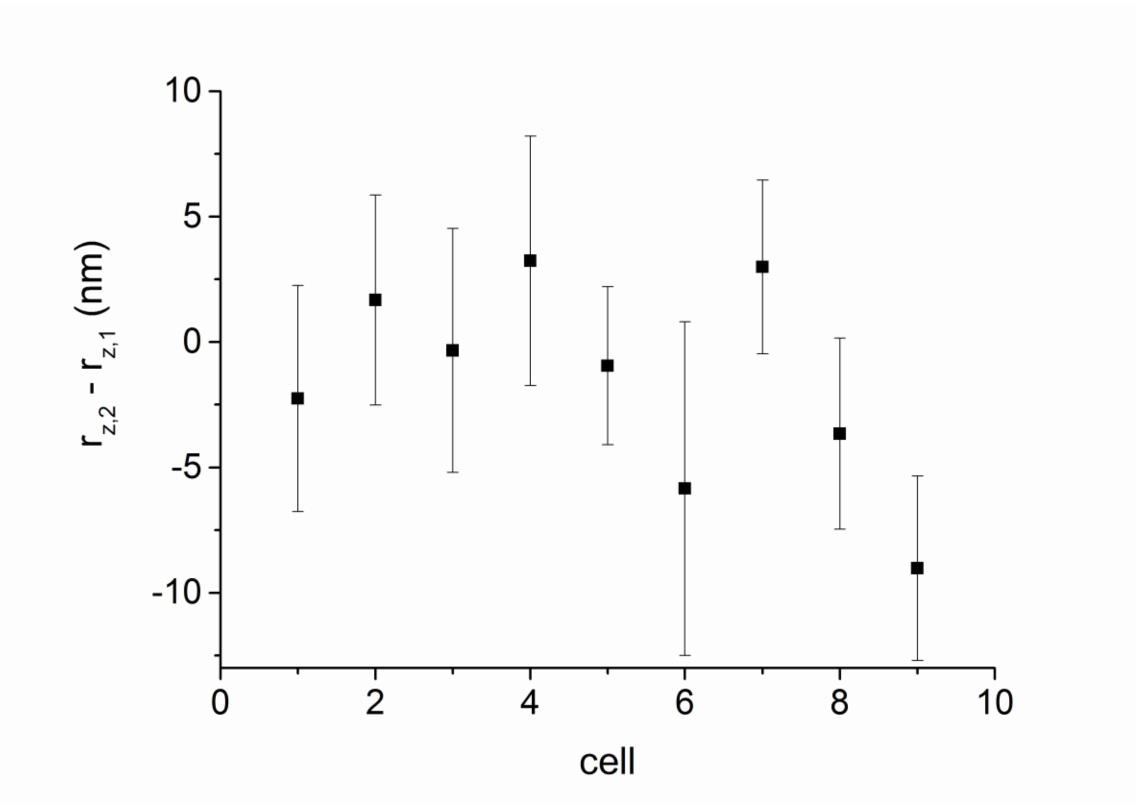

Fig. S4 – Effect of photodamage on the radius of the Z-ring. For 9 different cells for which most frames had sufficient contrast we compute the difference between the ring radius averaged over the first 10 sec of the experiment (first 15 frames), $r_{z,1}$, and that from the next 10 sec of the experiment (next 15 frames), $r_{z,2}$ (squares). The error bars were obtained by dividing the corresponding standard deviations to $\sqrt{N_i}$, $i=1,2$ (N_i is the number of frames used for averaging, $N_i<15$, since a few of the 15 frames had insufficient contrast). The data indicates that for most cells, $r_{z,2}-r_{z,1}=0$, within the experimental error.

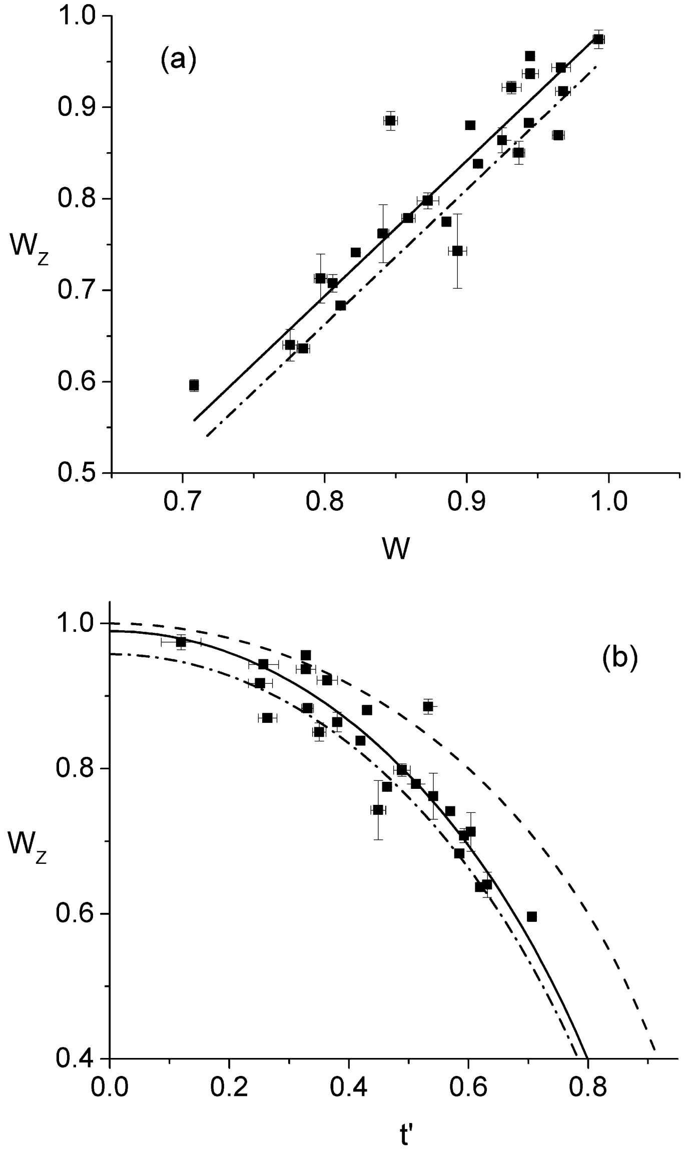

Fig. S5 – Assuming that FM4-64 stains the outer rather than the inner cell membrane does not significantly affect the observed Z-ring constriction dynamics. a) Same as in Fig. 3, only here the values of the measured cell radii, R and r, were reduced by the inter-membrane distance (16 nm). The best linear fit, $W_z = a_1 W + b_1$, to this data is obtained for $a_1 = 1.48 \pm 0.02$ and $b_1 = -0.49 \pm 0.01$ (full line). For comparison, the linear fit of Fig. 3 is also shown (dot-dashed line). b) Same as in Fig. 4, only here the measured cell radii were reduced by the inter-membrane distance. The theoretical model (full line) is obtained from the fit in a) and Eq. (1). It is shown together with the $W_z(t')$ theoretical curve from Fig. 4 (dot-dashed line) and the constriction dynamics of Eq. (1), $W(t')$, (dashed line).

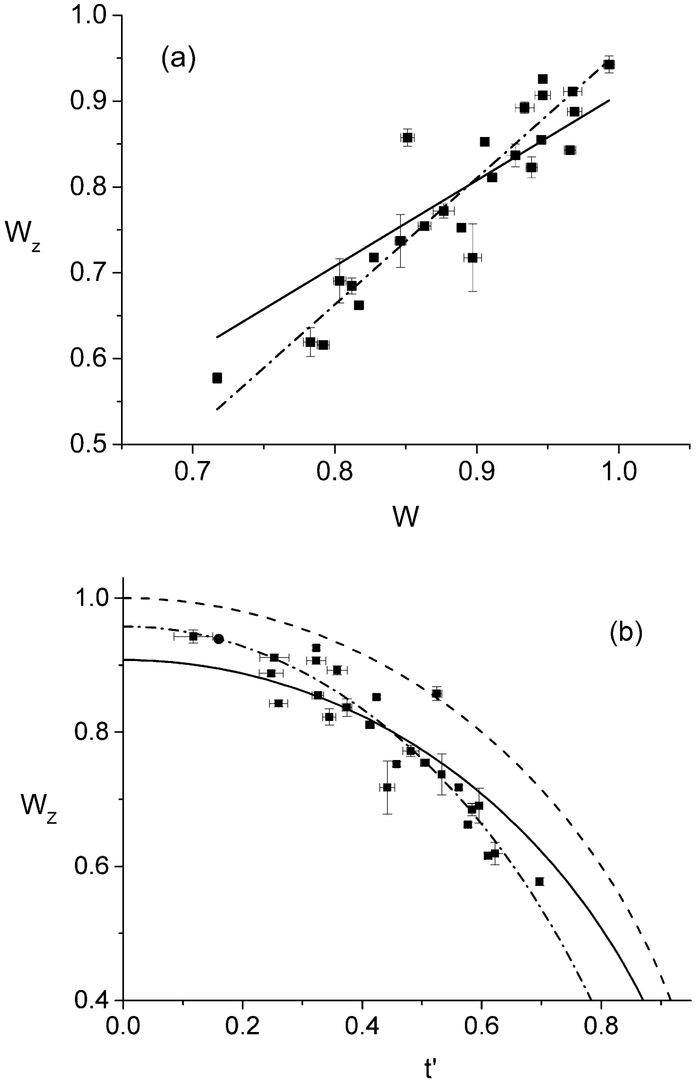

Fig. S6 – The Z-ring constriction dynamics is poorly described using a Z-ring model with a constant radial width. a) Same as in Fig. 3, only here the linear fit to the $W_z(W)$ data is constrained to have unit slope, $W_z = W + c$ (full line). The best fit is obtained for $c = -0.092 \pm 0.001$ corresponding to $R^2 = 0.79$ ($R^2 \equiv$ coefficient of determination). For comparison, the linear fit of Fig. 3 is also shown (dot-dashed line). It corresponds to a significantly larger value of R^2, $R^2 = 0.87$. b) Same as in Fig. 4, only here the theoretical model (full line) is obtained from the fit in a), corresponding to a constant radial width Z-ring. It is shown together with the $W_z(t')$ curve of Fig. 3 (dot-dashed line), corresponding to a Z-ring with a growing radial width, and the constriction dynamics of Eq. (1), $W(t')$, (dashed line).
